# Lido-OH, a Hydroxyl Derivative of Lidocaine, Produced a Similar Local Anesthesia Profile as Lidocaine With Reduced Systemic Toxicities

**DOI:** 10.3389/fphar.2021.678437

**Published:** 2021-09-16

**Authors:** Qinqin Yin, Weiyi Zhang, Bowen Ke, Jin Liu, Wensheng Zhang

**Affiliations:** ^1^ Department of Anesthesiology, West China Hospital, Sichuan University, Chengdu, China; ^2^ Laboratory of Anesthesia and Critical Care Medicine, Translational Neuroscience Centre and Sichuan Engineering Laboratory of Transformation Medicine of Anesthesiology, West China Hospital, Sichuan University, Chengdu, China; ^3^ Translational Neuroscience Centre, West China Hospital, Sichuan University, Chengdu, China

**Keywords:** lidocaine, toxicity, efficacy, hydroxyl derivatives, local anaestesia

## Abstract

**Background:** lidocaine is one of the most commonly used local anesthetics for the treatment of pain and arrhythmia. However, it could cause systemic toxicities when plasma concentration is raised. To reduce lidocaine’s toxicity, we designed a hydroxyl derivative of lidocaine (lido-OH), and its local anesthesia effects and systemic toxicity *in vivo* were quantitively investigated.

**Method:** the effectiveness for lido-OH was studied using mouse tail nerve block, rat dorsal subcutaneous infiltration, and rat sciatic nerve block models. The systemic toxicities for lido-OH were evaluated with altered state of consciousness (ASC), arrhythmia, and death in mice. Lidocaine and saline were used as positive and negative control, respectively. The dose-effect relationships were analyzed.

**Results:** the half effective-concentration for lido-OH were 2.1 mg/ml with 95% confident interval (CI95) 1.6–3.1 (lidocaine: 3.1 mg/ml with CI95 2.6–4.3) in tail nerve block, 8.2 mg/ml with CI95 8.0–9.4 (lidocaine: 6.9 mg/ml, CI95 6.8–7.1) in sciatic nerve block, and 5.9 mg/ml with CI95 5.8–6.0 (lidocaine: 3.1 mg/ml, CI95 2.4–4.0) in dorsal subcutaneous anesthesia, respectively. The magnitude and duration of lido-OH were similar with lidocaine. The half effective doses (ED_50_) of lido-OH for ACS was 45.4 mg/kg with CI95 41.6–48.3 (lidocaine: 3.1 mg/kg, CI95 1.9–2.9), for arrhythmia was 16.0 mg/kg with CI95 15.4–16.8 (lidocaine: 3.0 mg/kg, CI95 2.7–3.3), and for death was 99.4 mg/kg with CI95 75.7–124.1 (lidocaine: 23.1 mg/kg, CI95 22.8–23.4). The therapeutic index for lido-OH and lidocaine were 35.5 and 5.6, respectively.

**Conclusion:** compared with lidocaine, lido-OH produced local anesthesia at similar potency and efficacy, but with significantly reduced systemic toxicities.

## Introduction

Local anesthetics (LAs) are very critical in pain management. LAs inhibit the conduction of afferent nerve impulses of nociceptive stimuli, thus producing reliable analgesia while avoiding the common side effects of other analgesics such as gastro-intestinal bleeding, coagulation disorder, liver or kidney function impairment, addiction, tolerance, etc., (Ma et al., 2017; Morrison et al., 2021). However, LAs carry the risk of systemic toxicities, known as local anesthetic systemic toxicity (LAST), which frequently interferes with the central nervous system and the cardiovascular system, resulting in altering consciousness state, cardiovascular depression, arrhythmia, seizure, and asystole in extreme cases (Wadlund, 2017). LAST could be a serious problem for the elderly, pregnant women, fragile patients, or patients with impaired liver or kidney function. The risk of LAST increases in scenarios involving richly-vascularized tissue injection, inadvertent intravascular injection, prolonged continuous intravenous infusion, and injection of large volumes (Sagie and Kohane, 2010; Torp and Simon, 2010; EI-Boghdadly et al., 2018; Gitman and Barrington, 2018) Despite the ultrasound guided regional anesthesia technique, there is no evidence suggesting that the incidence of LAST has dropped to a clinically acceptable level so far.

To expand the application of LAs, LAST needs to be reduced. LAST is the results of LAs’ intrinsic pharmacological activity and chemo-physical properties. When plasma concentrations of LAs are elevated to a certain level, LA molecules accumulate in target organs such as the brain and heart. Due to LAs’ non-selective inhibition of ion channels, electrical activity in the neurons and myocardium are disturbed. Both lipo-solubility and interaction with ion channels are important for LAST.

Lidocaine is the most common LA used in clinic. Lidocaine molecules interact with sodium channels with the alky group from the amine end, by insertion of the alky group into the space between phenylalanine from DIV-S6 and leucine from DIII-S6 of sodium channel in the opening state. Modifying the alky group at the amine end of lidocaine may change the interaction with sodium channels. Lido-OH preserves the characteristic molecular structures of lidocaine, including the lipid aromatic group, the hydro amine group, and the amide linkage. Therefore, it may inherit the basic chemo-physical properties from lidocaine. Preserving anesthesia potency and reducing LAST might be well weighed by this modification in molecular structure.

The research hypothesis: 1) the potency and efficacy of analgesia for lido-OH were similar with lidocaine in mouse tail nerve block, rat dorsal subcutaneous infiltration, and rat sciatic nerve block models; 2) the doses for lido-OH to elicit central nerve toxicity, cardiac toxicity, and death were higher than lidocaine; and 3) less lido-OH, compared with lidocaine, is distributed into the brain and heart after intravenous injection.

## Materials and Methods

### Drugs and Materials

**FIGURE 1:**
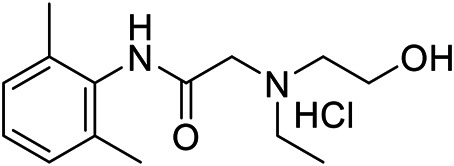
Molecular structure of lido-OH (C_14_H_22_N_2_O_2_).

Lido-OH hydrochloride (Figure 1) is a white powder with purity ≥99.5%. Lido-OH has logP of 1.28 and pKa of 6.91. It dissolves in saline with a pH of 6.0–6.5. Hydrochloride lidocaine solution at 2% (Human-well Pharmaceutical Ltd., Hubei, China) or lower concentrations (diluted with saline) were used. Solutions of lido-OH, lidocaine, and vehicle (0.9% saline) were prepared in the same day of the experiment.

### Animal Care

Animal relevant procedures in this study were conducted in accordance with the guide for the care and use of medical laboratory animals (Ministry of Health, China), and were supervised by the institutional animal experiment ethics committee of Sichuan University (Chengdu, China, Approval file No. 2015014A). Male Sprague-Dawley rats (Dossy Experimental Animal Company, Chengdu, China) aged 40–60 days, weighted 250–300 g were housed at 25°C with free access to food and water under 12 h light/12 h dark cycle. NIH mice (Dossy Experimental Animal Company, Chengdu, China) aged 30–40 days, weighted 25–40 g were housed at similar environment. Animals were acclimated to experimental environments and experimenters, handled daily to minimize stress-induced analgesia. Animals were randomized into groups. All behavioral tests were performed by experimenters who are blinded to the treatment each animal received.

### Local Anesthesia Effects

The local anesthesia effects of lido-OH were examined in the following three rodent models: mouse tail nerve block (mouse tail-flick test), rat dorsal skin infiltration anesthesia, and rat sciatic nerve block models. For each model, experiments were conducted in two steps with lidocaine and saline as positive and negative controls, respectively. Firstly, the concentration-response relationship was investigated; secondly, anesthesia intensity and time-course (onset time, action time) for lido-OH and lidocaine, at equipotent concentration or clinically relevant concentration (2%) were investigated.

Mouse tail-flick test(Lim et al., 2007): the distal end (3 cm from the tip) of a mouse tail was immersed in 50°C hot water. The time interval between the immersion and the flick of the tail was defined as the tail-flick latency, representing the tolerance for heat pain. Baseline for the tail-flick latency was measured before treatment, and mice with baselines <2 s were chosen. Unilateral 20 μl (40 μl total) lido-OH (0.5, 1.0, 1.5, 2.0, 4.0 and 6.5 mg/ml), lidocaine (2.0, 2.5, 3.0, 4.0 and 5.0 mg/ml), or saline were injected at 7 cm proximal to the tip of tail with 29-G syringes (*n* = 10). The tail-flick latency was tested at 5, 10, 30, 60, 90, and 120 min after injection, and then every hour afterward until tail-flick latency returned to baselines. Effective and complete tail nerve block was defined when tail-flick latency was ≥4 s and ≥6 s, respectively. A cut-off time was set to 6 s to avoid burning injury.

Rat dorsal skin infiltration: 100 μL lido-OH (1.25, 2.5, 5.0, 7.5, 10.0 and 20.0 mg/ml), lidocaine (1.25, 2.5, 5.0, 7.5, 10.0 and 20.0 mg/ml), or saline was subcutaneously injected at the left dorsal region (*n* = 8–10). Then six pinpricks were made with a blunt 18-gauge needle attached to a 26-g von-frey filament at an interval of 15 s. Cutaneous trunci muscle reflex (pinprick elicits skin movement over the back, abbreviated as CTMR) was observed; effective skin infiltration anesthesia was defined when more than three out of six CTMR were inhibited, and no CTMR should be observed in complete anesthesia. CTMR was tested at 5, 10, 30, 60, 90, and 120 min after injection.

Rat sciatic nerve block (I and DS, 2010; Templin et al., 2015; Yin et al., 2016; Zhang et al., 2017): 0.2 ml of lido-OH (7.0, 7.7, 8.4 and 10.1 mg/ml), lidocaine (6 mg/ml, 8 mg/ml), or saline were injected near the left sciatic nerve in isoflurane-sedated SD rats (*n* = 8). After injection, the rats were allowed to recover freely in a transparent cage with free access to food and water. Sensory and motor function of the injected leg were tested with modified hot plate test (RB-200 Hot Plate, Chengdu Techman Software Co., Ltd.) and postural thrust test, respectively, at 5, 10, 30 min, 1, 2, 4 h after injection, and every 2 h afterward until the test value returned to baselines as tested before the experiment. In the revised hot plate test, the paw of the injection side was placed on a 56°C-metal plate, the time interval between placement and withdrawal of paw was defined as paw withdrawal latency (PWL). In postural thrust test, a rat was vertically held to place the paw stand against an electronic balance, so that the leg muscle strength could be measured as grams displayed by the balance. The degree of sensory and motor block was evaluated by maximum possible effect (MPE) calculated as the following:

$$\mathrm{MPE}=\frac{\text{Test value}-\text{baseline}}{\text{Cutoff value }-\text{baseline}}\text{∗}100\text{\%}$$

Effective and complete anesthesia was considered as MPE >50% and >90%, respectively. The cut-off PWL was set at 12 s to avoid tissue injury.

### Systemic Toxicity

Systemic toxicity was investigated in mice with the same dose-ranges used in these experiments, set according to preliminary studies.

Central nervous system toxicity (Cheung et al., 2011): Mice received a single injection of lido-OH or lidocaine through the tail vein using 0.3 ml insulin syringe and 30G hypodermic needle (total injection volume <0.2 ml, injection speed at 0.1 ml/s), then the mice were removed to a transparent plastic box and observed for behaviors of altered state of consciousness (ASC) such as convulsion, ataxia, sedation, or loss of righting reflex. The intravenous doses of lido-OH used in the systemic toxicity experiment were: 33.3, 46.7, 53.3, and 71.1 mg/kg; those of lidocaine were 2.7, 3.0, 3.6, and 4.7 mg/kg (*n* = 10).

Electrocardiography study (Cheung et al., 2011): Mice inhaled with 1.5% isoflurane and oxygen via a custom-made plastic transparent mask. Then theelectrocardiogram (ECG) was recorded (BL-420, Chengdu Techman Software Co., Ltd.) with three metal subcutaneous hook-electrodes. After 5 min of isoflurane anesthesia, stable heart rate (HR), P-R interval, and the wave form of QRS complex were recorded as baselines. Then lidocaine (2.0 mg/kg, 2.5 mg/kg, 3.0 mg/kg and 3.5 mg/kg), lido-OH (15 mg/kg, 16.7 mg/kg, 20 mg/kg and 25 mg/kg) or saline were injected into tail vein (*n* = 5). HR, P-R interval and QRS wave length after the injection were recorded and analyzed. Over 30% change of HR, P-R interval, or QRS wave length from the baselines were considered evidences for cardiac toxicity.

Death: Mice received single tail vein injection of lidocaine (1.6, 11.2, 20.8, 30.4, and 40.0 mg/kg) or lido-OH (51.2, 80.9, 110.7, 140.4, and 170 mg/kg, *n* = 10). Death was defined when asystole and apnea lasted for at least 5 min.

All animals that received local application of drugs were inspected for at least 24 h for signs of systemic drug distribution, such as analgesia for paralysis on the un-injected leg; systemic toxicity such as convulsion, ataxia, sedation, or loss of righting reflex; and macroscopic local tissue reaction such as skin rash, scratches, hematoma, ulcer, blisters, etc.

### Tissue Toxicity

The tissue toxicity of lido-OH and lidocaine for peri-sciatic nerve block was evaluated by histological examinations. Seven days after the sciatic nerve block experiments, after sacrificing rats with overdose of intravenous Propofol, the sciatic nerve with surrounding tissue was harvested, and stained with Hematoxylin-Eosin (HE) for histological examination. The degree of local tissue toxicity was semi-quantitated and measured with a 0–4 scoring system: 0 = normal; 1 = 0–25% of area involved; 2 = 25–50% of area involved; 3 = 50–75% involved; and 4 = 75–100% involved. The histological examination was performed by a pathologist who was blinded to the treatment (Shackelford et al., 2002)

### Statistical Analysis

Data are presented as mean ± standard deviation unless indicated otherwise. Kolmogorov-Smirnov test was used for normal distribution of data. Repeated-measurements of ANOVA and Kaplan-Meiser analysis were used to analyze tail-flick test data. The onset time and duration of anesthesia was analyzed with one-way ANOVA. Statistical analysis and was performed and figures were generated using PRISM (Version 7 for Mac OS X) or Excel (Microsoft Excel 2016 for MAC). The dose-response curves were generated in PRISM using variable slope model fitting with least square. Potency parameters including 5%-effective dose/concentration (ED_05_/EC_05_), half-effective dose/concentration (ED_50_/EC_50_) and 95%-effective dose/concentration (ED_95_/EC_95_) with their 95% confident intervals (CI_95_) were calculated using Probit Analysis in SPSS Statistics (IBM SPSS Statistics Version 25 for Mac OS), except that the EC_50_ for lidocaine in rat sciatic nerve block was measured by the Up-and-Down method (Cheung et al., 2011). Difference was considered significant when *p* < 0.05.

## Results

### Local Anesthesia

Both lido-OH and lidocaine produced reversible, concentration-dependent local anesthesia including tail nerve block, skin infiltration anesthesia, and sciatic nerve block. The EC_50_ of lido-OH was close to that of lidocaine; the analgesia magnitude and action duration time were similar between lido-OH and lidocaine in tail nerve block and dorsal skin infiltration anesthesia. However, lido-OH exerted sciatic nerve block that lasted for a shorter time, and was less intense than lidocaine. No anesthesia effect was observed for saline.

**FIGURE 2:**
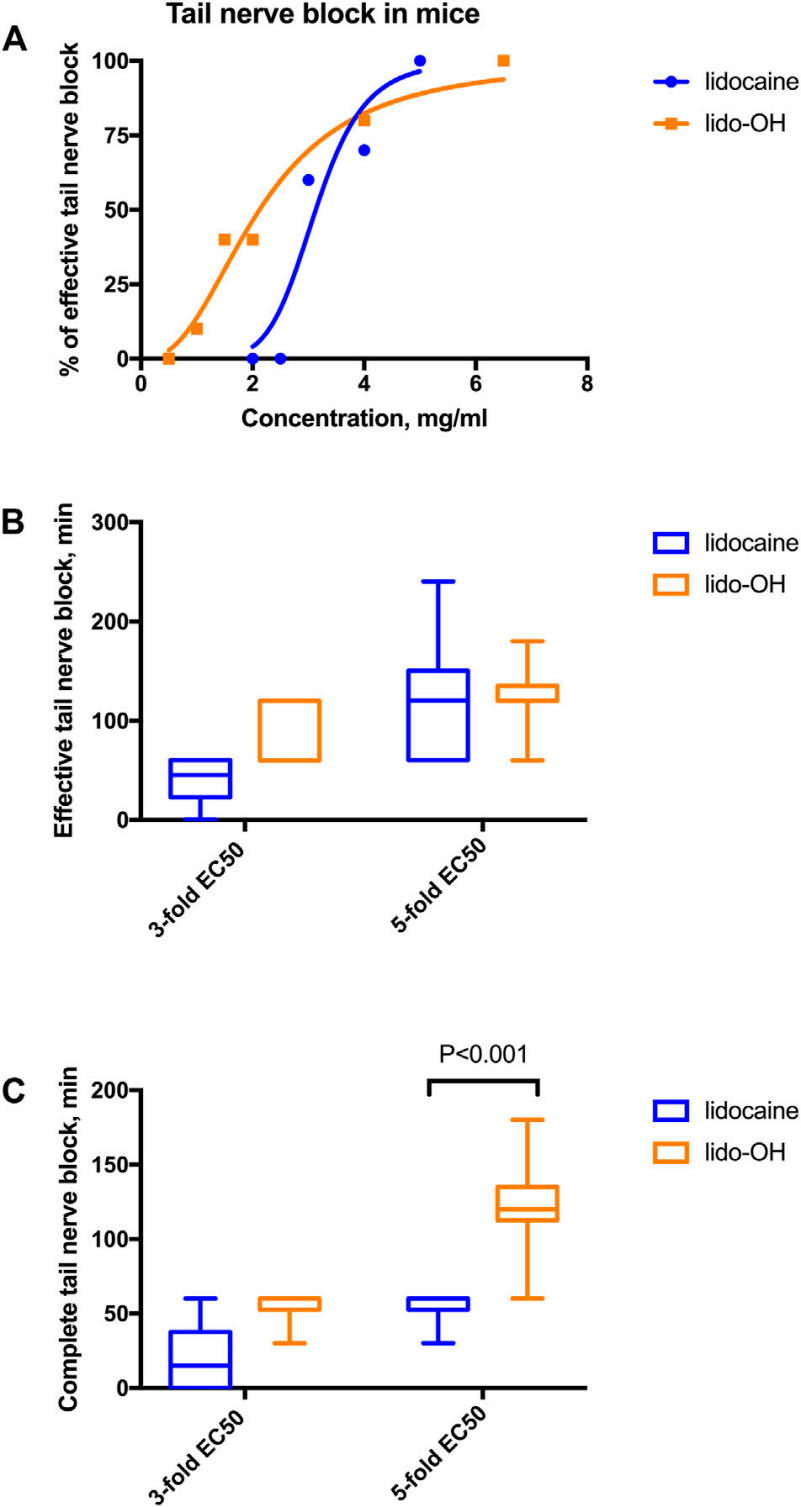
The action of lido-OH and lidocaine in mice tail nerve block (n = 10): The concentration-response curve **(A)**; duration of effective **(B)** and complete **(C)** tail nerve block. EC_50_ for lidocaine: 3.1 mg/ml, EC_50_ for lido-OH: 2.1 mg/ml.

Tail nerve block effectiveness increased with concentration escalated for both lidocaine and lido-OH. The concentration-effectiveness curve was steeper for lidocaine than lido-OH (Figure 2A). The EC_50_ for lido-OH was 2.1 mg/ml (95% confidence interval, CI_95_: 1.6–3.1), while it is 3.1 mg/ml for lidocaine (CI_95_: 2.6–4.3). At 3-fold EC_50_, there was no difference of duration of action between lido-OH and lidocaine (for effective tail nerve block, 80 ± 31min vs. 40 ± 24min, *p* = 0.18, for complete block, 55 ± 12 min vs. 20 ± 24 min, *p* = 0.041). (Figure 2B). At 5-fold of EC_50_, the duration of effective anesthesia for lido-OH and lidocaine is of no statistical difference. (126 ± 34 min vs. 120 ± 66 min, *p* = 0.81), but the complete block duration for lido-OH was longer than lidocaine (123 ± 34 min vs. 55 ± 12 min, *p* < 0.001, Figure 2C).

**FIGURE 3:**
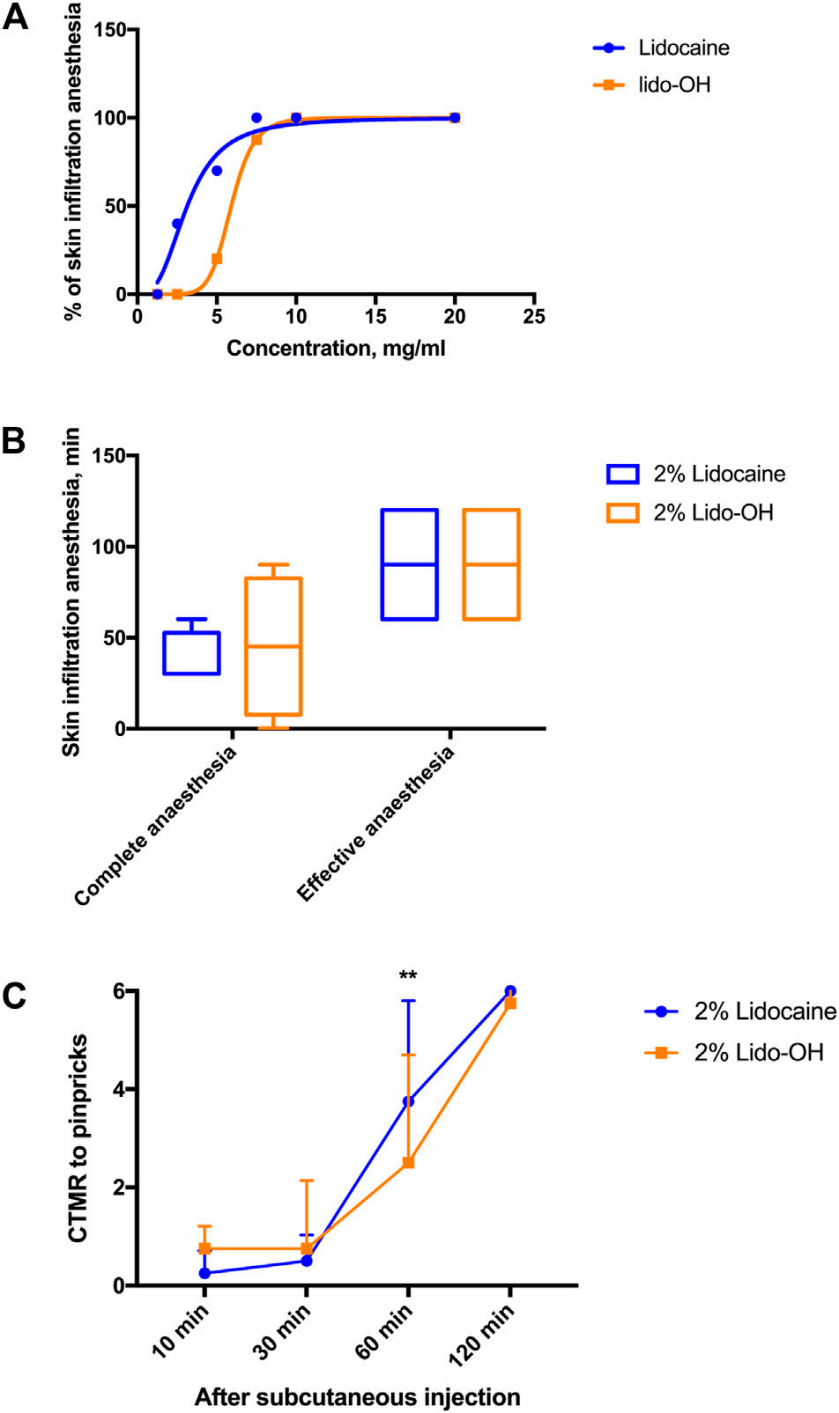
The actions of lido-OH and lidocaine in rat subcutaneous anesthesia (n = 8–10): the concentration-effectiveness curves **(A)**; the duration of effective and complete skin infiltration anesthesia **(B)** and the magnitude of anesthesia evaluated by cutaneous trunci muscle reflex (CTMR) to pinpricks **(C)**. **: *p* = 0.0041. The dotted line represents at each sampling time the total number of pinpricks, which are also the CTMR numbers for saline.

There was an apparent concentration-dependent manner for lido-OH and lidocaine in the rat infiltration anesthesia model. The concentration-response curve for lidocaine seemed steeper than lido-OH (Figure 3A). The EC_50_ for lido-OH in rat subcutaneous infiltration anesthesia was 5.9 mg/ml (CI_95_: 5.8–6.0), compared with 3.1 mg/ml for lidocaine (CI_95_: 2.4–4.0). At 10 mg/ml, both drugs elicited 100% effective skin infiltration anesthesia. At 20 mg/ml (2%), there was no statistic difference of anesthesia duration between lido-OH (45 min, interquartile range, IQR: 7.5–82.5) and lidocaine (30 min, IQR: 30–52.5, *p* = 0.86, Figure 3B); although the magnitude of subcutaneous anesthesia by lido-OH at the recovering period (the 60-min post-injection time point) was greater than that of lidocaine (*p* = 0.0041, Figure 3C).

**FIGURE 4:**
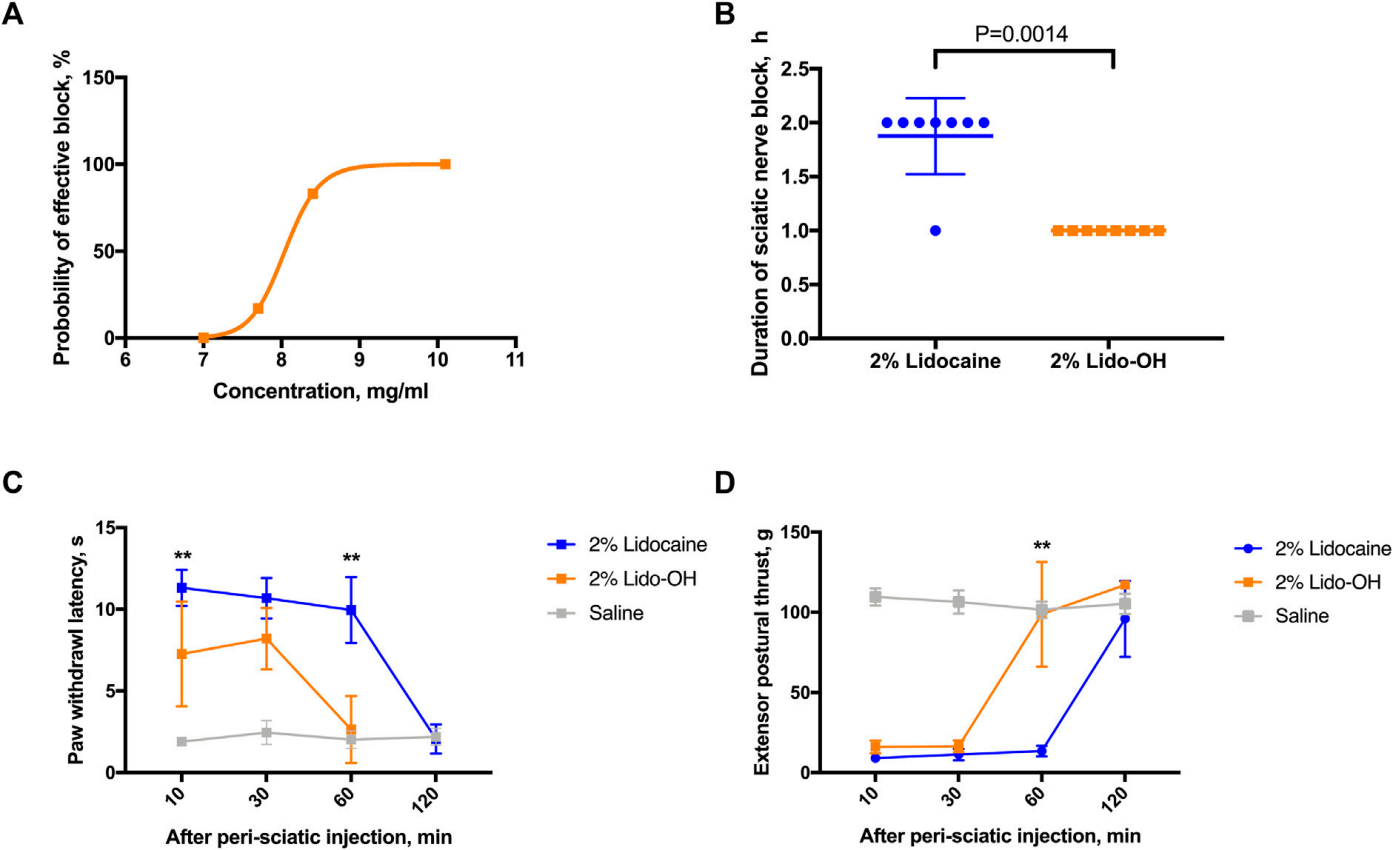
The effects of lido-OH and lidocaine in rat sciatic nerve block (*n* = 8): The concentration-effectiveness curve of lido-OH **(A)**; the duration of sciatic nerve block **(B)**; the magnitude of sensory blockade evaluated by prolonged paw withdrawal latency to noxious heat **(C)**; and the magnitude of motor blockade measured by reduction of extensor postural thrust **(D)**. **: *p* < 0.01.

In the rat sciatic nerve block model, there is also an obvious concentration-effectiveness relationship for lido-OH (Figure 4A). The EC_50_ for lido-OH here was 8.2 mg/ml (CI_95_: 8.0–9.4), compared with 6.9 mg/ml for lidocaine (CI_95_: 6.8–7.1, calculated by the up-and-down method, SI Supplementary Table S1). 100% Effective sciatic nerve block was achieved with lido-OH at 10.1 mg/ml (1%). At 2%, lido-OH produced action time shorter than lidocaine (1 ± 0 h *vs*. 1.9 ± 0.4 h, *p* = 0.0014, Figure 4B), the block intensity was less compared with lidocaine (Figures 4C,D).

**FIGURE 5:**
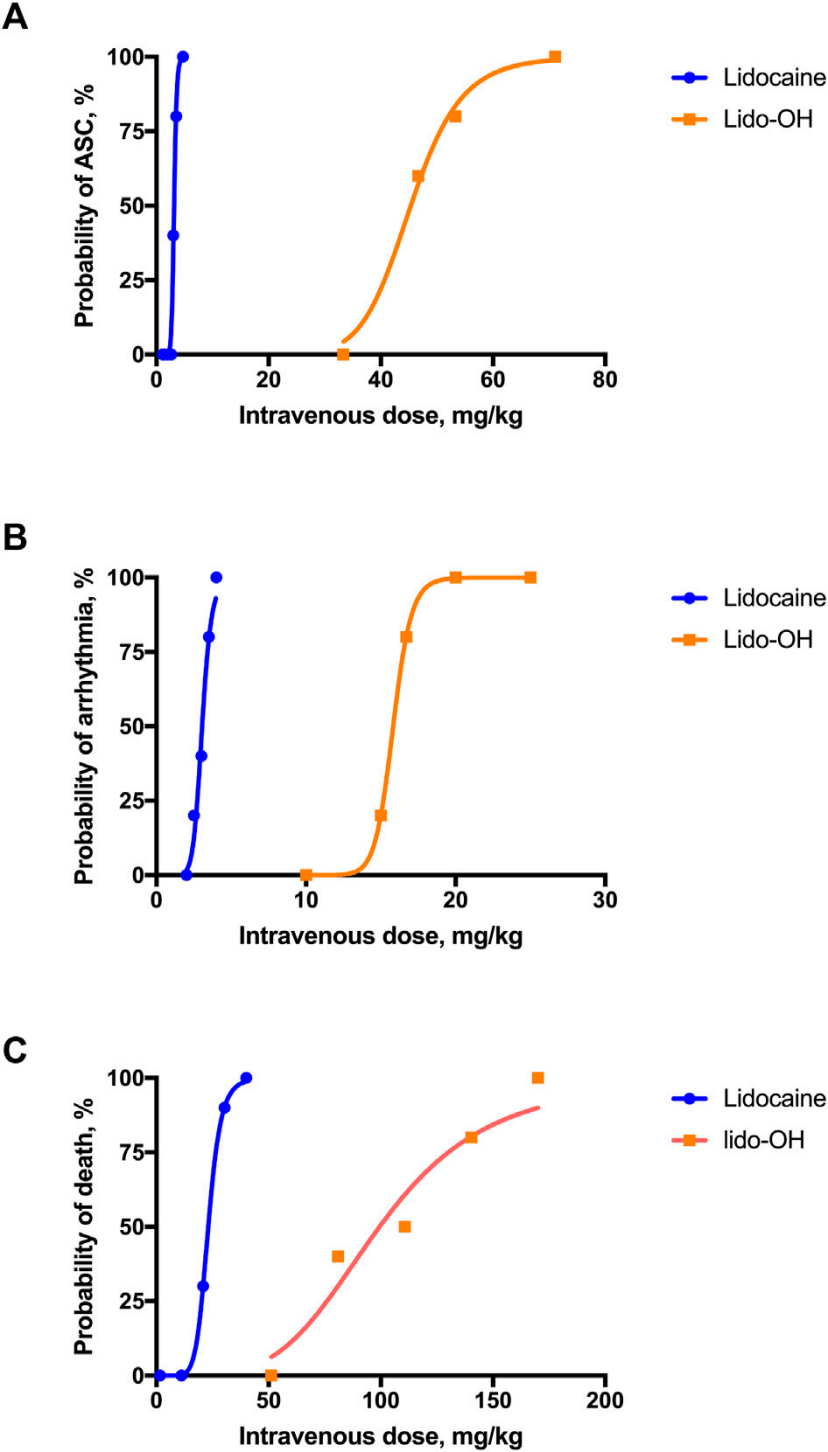
Systemic toxicities were produced by single intravenous injection of lidocaine or lido-OH. Dose-toxicities relationships were evaluated in altered state of consciousness **(A)**, *n* = 10), arrhythmia **(B)**, *n* = 5), and death **(C)**, *n* = 10). ASC: altered state of consciousness.

For lido-OH and lidocaine, dose-dependent ASC, arrhythmia, and death were observed for both lidocaine and lido-OH. The dose-toxic response curve for lidocaine was obviously steeper than lido-OH, except for arrhythmia (Figure 5). The ED_50_ for lido-OH to produce ASC, arrhythmia, and death were 14-fold, 5-fold, and 4-fold greater than those for lidocaine, respectively (Table 1). The therapeutic index in mice, determined by ED_50_ in tail nerve block (ED_50_ transferred from EC_50_ assuming body weight of 30 g) divided by LD_50_, was 35.5 and 5.6 for lido-OH and lidocaine, respectively. The LD_05_/ED_95_ were 8.0 and 2.5 for lido-OH and lidocaine, respectively.

**TABLE 1:** Equivalent doses for central nervous system toxicities, cardiac toxicities, death, and tail nerve block in mice.

Adverse events	Parameters	Lidocaine, mg/kg	Lido-OH, mg/kg
ASC	ED_05_ (CI95)	2.6 (1.9–2.9)	36.6 (18.9–41.7)
	ED_50_ (CI95)	3.1 (3.0–3.3)	45.4 (41.6–48.3)
	ED_95_ (CI95)	3.87 (3.5–4.7)	58.24 (52.6–91.0)
Arrhythmia	ED_05_ (CI95)	2.3 (1.8–2.6)	14.8 (12.8–15.4)
	ED_50_ (CI95)	3.0 (2.7–3.3)	15.8 (15.8–15.9)
	ED_95_ (CI95)	3.9 (3.5–5.0)	17.3 (16.6–20.3)
Death	LD_05_ (CI95)	15.5 (2.9–19.6)	57.7 (31.6–72.9)
	LD_50_ (CI95)	23.1 (22.8–23.4)	99.4 (75.7–124.1)
	LD_95_ (CI95)	31.9 (27.9–44.3)	172.3 (141.1–280.8)

The development of toxic symptoms in conscious mice was dose-related (SI Supplementary Figure S1). For intravenous lidocaine, there was no obvious systemic toxic behaviors if the injection dose was lower than 3.6 mg/kg. At 3.6–11.2 mg/kg, toxic symptoms included ataxia, sedation, convulsion, and LORR. As doses increased within this range, the incidences and durations of these symptoms increased, and the onset times shortened. As intravenous lidocaine increased to 20.8–40 mg/kg, LORR developed immediately, followed by apnea, and finally caused death (SI Supplementary Table S2). For lido-OH, there were no systemic toxic symptoms developed <46.7 mg/kg. At 46.7–53.3 mg/kg, toxic symptoms began to developed, including ataxia, convulsion, and LORR. The duration of toxic symptoms prolonged as the dosage escalated. As intravenous doses increased above 71.1 mg/kg, mice had LORR and apnea. Most of mice with apnea died, only two mice recovered (SI Supplementary Table S3). The incidence of apnea and death increased with increasing of dosage.

Intravenous injection of lidocaine or lido-OH induced arrhythmia, as intravenous dose increased, heart rate decreased, QRS-complex length widened, and P-R interval prolonged (SI Supplementary Figure S2). The extent of ECG changes is also dose-dependent (SI Supplementary Figure S3). There was no death in lido-OH injected animals (*n* = 20), nor other systemic toxic behaviors. Of mice that received intravenous lidocaine (*n* = 20), three died of irreversible apnea (two mice, 2.5 mg/kg and 3.5 mg/kg, respectively) or asystole (one mouse, 3.5 mg/kg); moreover, four mice developed short-lasting apnea (5–40 s, for 2.5 mg/kg and 3.5 mg/kg lidocaine), and one mouse had convulsion (18 s, for 2.0 mg/kg lidocaine).

Local tissue toxicity: After local application of drugs, there was no evidence observed for numbness or paralysis in the un-treated region, no systemic toxic behaviors, or local tissue toxicity (such as skin rash, scratches, hematoma, ulcer, blisters, etc.). The gross appearance of tissues was normal. In histological examination of the sciatic nerve and surrounding tissues in light microscope, there was no signs for necrosis or severe inflammation. The histological evaluation scores for lidocaine (0.6 ± 0.1), lido-OH, and saline were similar (*p* = 0.0862).

## Discussion

Lido-OH has local anesthesia property similar to lidocaine. The half effective concentration for lido-OH and lidocaine were close in three local anesthesia models. The efficacy of lido-OH was similar in terms of anesthesia intensity and action time, except sciatic nerve block, where lido-OH was half as long as that for lidocaine. More importantly, lido-OH has less systemic toxicity than lidocaine. Lido-OH’s ED_50_ for central nervous system toxicity, arrhythmia, and death were14-fold, 5-fold, and 4-fold greater than those for lidocaine, respectively. The therapeutic index for lido-OH was 35.5, approximately 7 times greater than lidocaine. The dose-response curves for lido-OH were less steep than those for lidocaine.

The anesthetic potency and efficacy of lido-OH were consistent with its physiochemical properties. The modification of lidocaine molecular structure was small and simple, only a hydroxyl group was connected to the amine end. Therefore, there was slight change of physiochemical properties. Two important physiochemical parameters of lido-OH, logP (representing hydrophobicity) and pKa (reflexes non-ionized vs. Ionized molecules under physiological pH) slightly decreased compared with lidocaine, which means that lido-OH was less lipid-soluble, and has greater fraction of extracellular non-ionized molecules. According to the structure activity relationship, local anesthesia potency, duration and onset of action are determined by lipid solubility and pKa. This may explain the relatively shorter duration and lower intensity in sciatic nerve block for lido-OH, since the blockade of the largest peripheral nerve requires plenty of non-ionized drug molecules to penetrate surrounding tissues and thick nerve sheath. In tail nerve block and skin infiltration, on the other hand, there is less lipid tissue that drug molecules have to penetrate, hence lido-OH has demonstrated similar or even greater effectiveness compared to lidocaine. In general, the local anesthesia effects could be explained by the structure-activity theories of local anesthetics.

Lido-OH has demonstrated profound reduction in systemic toxicity in comparison to lidocaine. The reduction of systemic toxicity was not only reflected by increased LD_50_ or ED_50_ for ASC and arrhythmia, but also seem in the slope of dose-toxic response curves, therapeutic index, and the ratio of LD_05_/ED_95_. Particularly for central nervous system toxicity, the ED_50_ of lido-OH for ACS was over 10-fold greater than lidocaine. Even under isoflurane-sedation, mice receiving lido-OH did not developed convulsion, whereas those receiving lidocaine did. It seems that the synthetic toxicity with isoflurane was greater for lidocaine, in forms of respiratory depression, convulsion, severe QRS wave widening, and death.

The reasons for the reduction in systemic toxicity remained unknown, however, multiple factors may have been involved. First, the physiochemical properties of amide local anesthetics. Lido-OH has lower lipid solubility, which could result in decreased toxicity. However, the extent of systemic toxicity reduction has far exceeded that of its lipophilicity. Second, the pharmacokinetic properties, such as different distribution in the brain or the heart, plasma protein binding, might also contribute to lido-OH’s safety. Given lido-OH’s obvious decreased impact in central nerve system and electrophysiological activity, it is possible that lido-OH may have reduced ability to diffuse through the brain-blood barriers, or have less affinity to the brain neurons, myocardium, or rhythmic cells in the heart. Third, the intrinsic pharmacological action, e.g. the intensity and selectivity of block to ion-channels, (EI-Boghdadly et al., 2018; Lirk et al., 2018), TNF-α induced protein kinase block, cell metabolism pattern, neuron electrophysiological properties, or the impact on ion channels responsible for action potential in myocardium, might be different between lido-OH and lidocaine (Gitman et al., 2019) Further research focusing on the above factors could be helpful in revealing the mechanisms for lido-OH’s reduction in systemic toxicity. It could be of benefit to understand the structure-activity relationship, in order to provide helpful experience in developing new local anesthetics with low LAST.

With the strategies such as ultrasound-guided technique, limitation of total drug dose, and co-application of vessel constrictors, the incidence of LAST seems to have decreased to a clinically non-important level, but is by no means unignorable (Gitman and Barrington, 2018) (Macfarlane et al., 2021) In addition, it is now more and more popular to use intravenous local anesthetics for intra-operative analgesia and chronic pain control (Vanstone and Rockett, 2016; Weibel et al., 2016; Song et al., 2017; Farahmand et al., 2018; Soto et al., 2018; Clivio et al., 2019), however, it may increase the incidence of LAST, because plasm drug concentration would increase more rapidly than local application (Gitman et al., 2019; Torp and Simon, 2020). A fundamental method to increase the safety of local anesthetics is to ensure that even if all drugs applied are accidentally injected into the circulation system, there could be a margin wide enough from the actual plasma concentration to the concentration responsible for the earliest toxicities. To achieve this goal, drugs with high therapeutic index will be needed. Lido-OH, hopefully, might be one of these drugs. It needs about 7.2 mg/kg (EC95: 5.4 mg/ml or 0.5%) for lido-OH to produce effective tail nerve block in 95% of mice, but requires 36.6 mg/kg (ED_05_ for ACS) intravenous lido-OH to cause symptoms in 5% of mice to exhibit central nervous toxic behaviors, the latter was 8-times greater than the former. Injection of lido-OH at 2%, about 4-fold of EC_95_, which was converted to 26.4 mg/kg, is unlikely to cause symptoms because it is still far below the intravenous doses for ACS. In fact, there was no behavioral evidence for LAST in mice treated with lido-OH < 46.7 mg/kg.

In this study, we mainly focused on effectiveness and safety properties, the pharmacokinetic properties were not involved. The impact of lido-OH on complex action potential in isolated nerves or ion-channels was not performed. Also, the route of drug application was confined to nerve block and skin infiltration, leaving topical anesthesia, epidural anesthesia, intrathecal anesthesia, and continuous infusion un-revealed. Systemic toxicity was evaluated by altering of consciousness state and arrhythmia, some other important aspects, such as hemodynamic stability, tidal volume, liver and kidney function, were not studied. Despite the above limitations, the basic local anesthesia effects and major systemic toxicities of lido-OH has been truthfully delineated.

In summary, we reported a lidocaine derivative, lido-OH with an extra hydroxyl group at the amine end, could produce local anesthesia effects similar to lidocaine, more importantly, with systemic toxicities several times smaller. In an era where local anesthetics play an essential role in pain management, lido-OH might have clinical value.

## Data Availability

The raw data supporting the conclusion of this article will be made available by the authors, without undue reservation.

## Abbreviation

Lido-OH, less toxic lidocaine derivative
