# Supplementary material for: Lido-OH, a Hydroxyl Derivative of Lidocaine, Produced a Similar Local Anesthesia Profile as Lidocaine With Reduced Systemic Toxicities
Source: Front Pharmacol. 2021 Sep 16;12:678437. doi: 10.3389/fphar.2021.678437 (PMC8481665; doi:10.3389/fphar.2021.678437)
Supplement: Supplementary file 1 [file DataSheet1.docx]

**Supplemental information**

**Table S1**. Determination of half effective concentration of lidocaine in sciatic nerve block in rats

| Dose (mg/ml) | $log(Dose)$ | Rat’s No. | | | | | | | | | |
| --- | --- | --- | --- | --- | --- | --- | --- | --- | --- | --- | --- |
|  |  | 1 | 2 | 3 | 4 | 5 | 6 | 7 | 8 | 9 | 10 |
| 8 | 0.9031 | + |  | + |  | + |  | + |  | + |  |
| 6 | 0.7782 |  | - |  | - |  | - |  | - |  | - |

**Table S2.** The incidence of systemic toxic symptoms after intravenous injection of lidocaine in mice

| Dose (mg/kg) | n | Ataxia | Convulsion | LORR | Sedation | Apnea | Death |
| --- | --- | --- | --- | --- | --- | --- | --- |
| 3.0* | 5 | 0 | 0 | 0 | 0 | 0 | 0 |
| 3.6 | 5 | 3 | 1 | 0 | 0 | 0 | 0 |
| 4.7 | 5 | 5 | 1 | 1 | 0 | 0 | 0 |
| 11.2 | 10 | 3 | 7 | 6 | 3 | 0 | 0 |
| 20.8 | 10 | 6 | 10 | 10 | 7 | 3 | 3 |
| 30.4 | 10 | 0 | 8 | 10 | 0 | 9 | 9 |
| 40.0 | 10 | 0 | 4 | 10 | 0 | 10 | 10 |

*: there were no systemic toxic symptoms observed for lower doses.

**Table S3.** The incidence of systemic toxic symptoms after intravenous injection of lido-OH in mice

| Dose (mg/kg) | n | Ataxia | Convulsion | LORR | Sedation | Apnea | Death |
| --- | --- | --- | --- | --- | --- | --- | --- |
| 46.7* | 5 | 3 | 0 | 0 | 0 | 0 | 0 |
| 51.2 | 10 | 4 | 1 | 2 | 0 | 0 | 0 |
| 53.3 | 5 | 8 | 0 | 0 | 8 | 0 | 0 |
| 71.1 | 5 | 4 | 3 | 5 | 0 | 1 | 1 |
| 80.9 | 10 | 6 | 9 | 10 | 3 | 3 | 4 |
| 110.7 | 10 | 4 | 9 | 10 | 0 | 3 | 5 |
| 140.4 | 10 | 3 | 5 | 10 | 0 | 7 | 8 |
| 170.7 | 10 | 0 | 3 | 10 | 0 | 10 | 10 |

*: there were no systemic toxic symptoms observed for doses < 46.7 mg/kg.


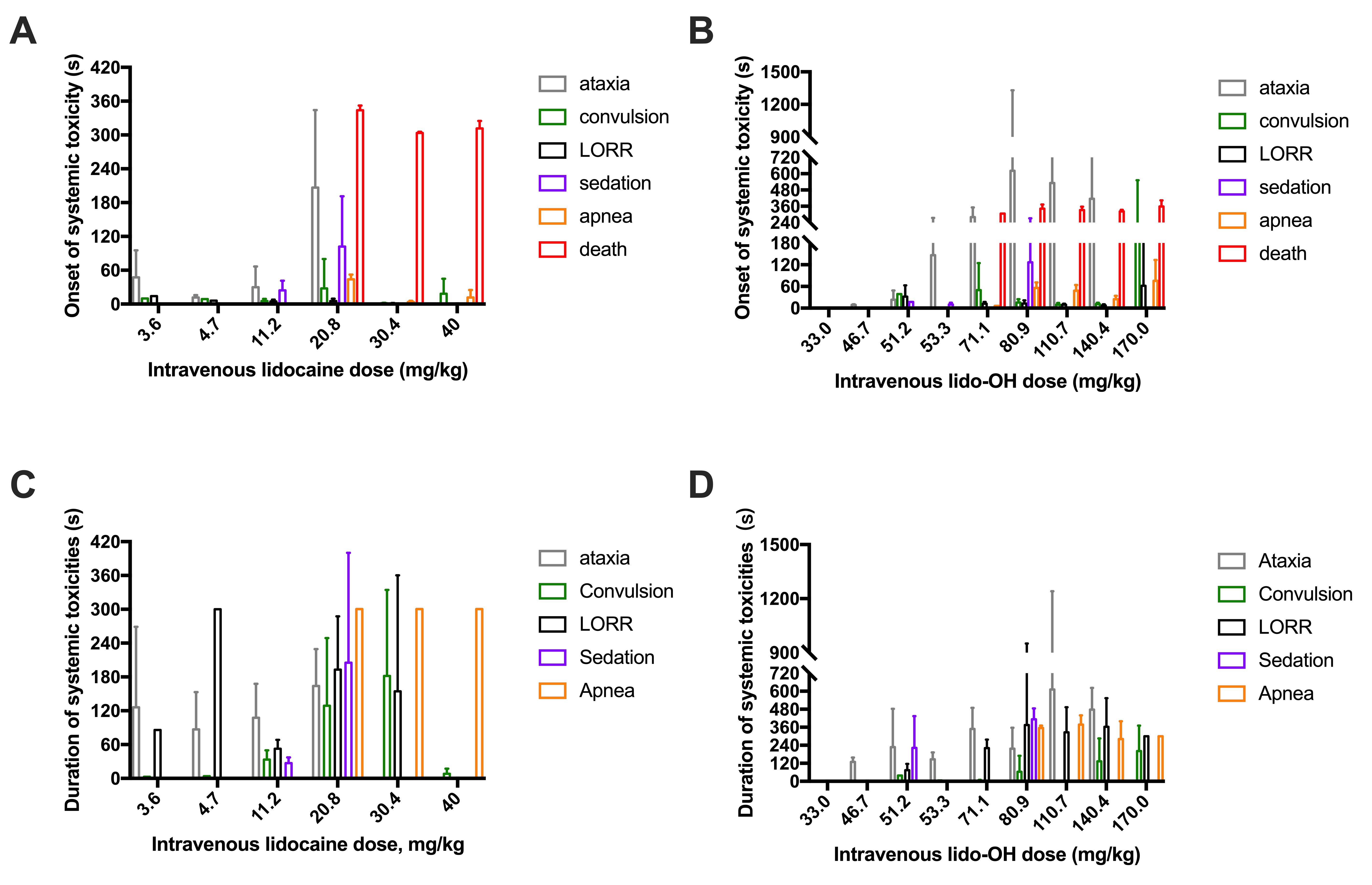


**Fig S1**. The onset and duration of symptoms after intravenous injection of lidocaine and lido-OH in mice. The onset of systemic toxicity symptoms after intravenous injection of lidocaine (A) or lido-OH (B); the duration of systemic toxicity symptoms after intravenous injection of lidocaine (C) or lido-OH (D). LORR: loss of righting reflex.


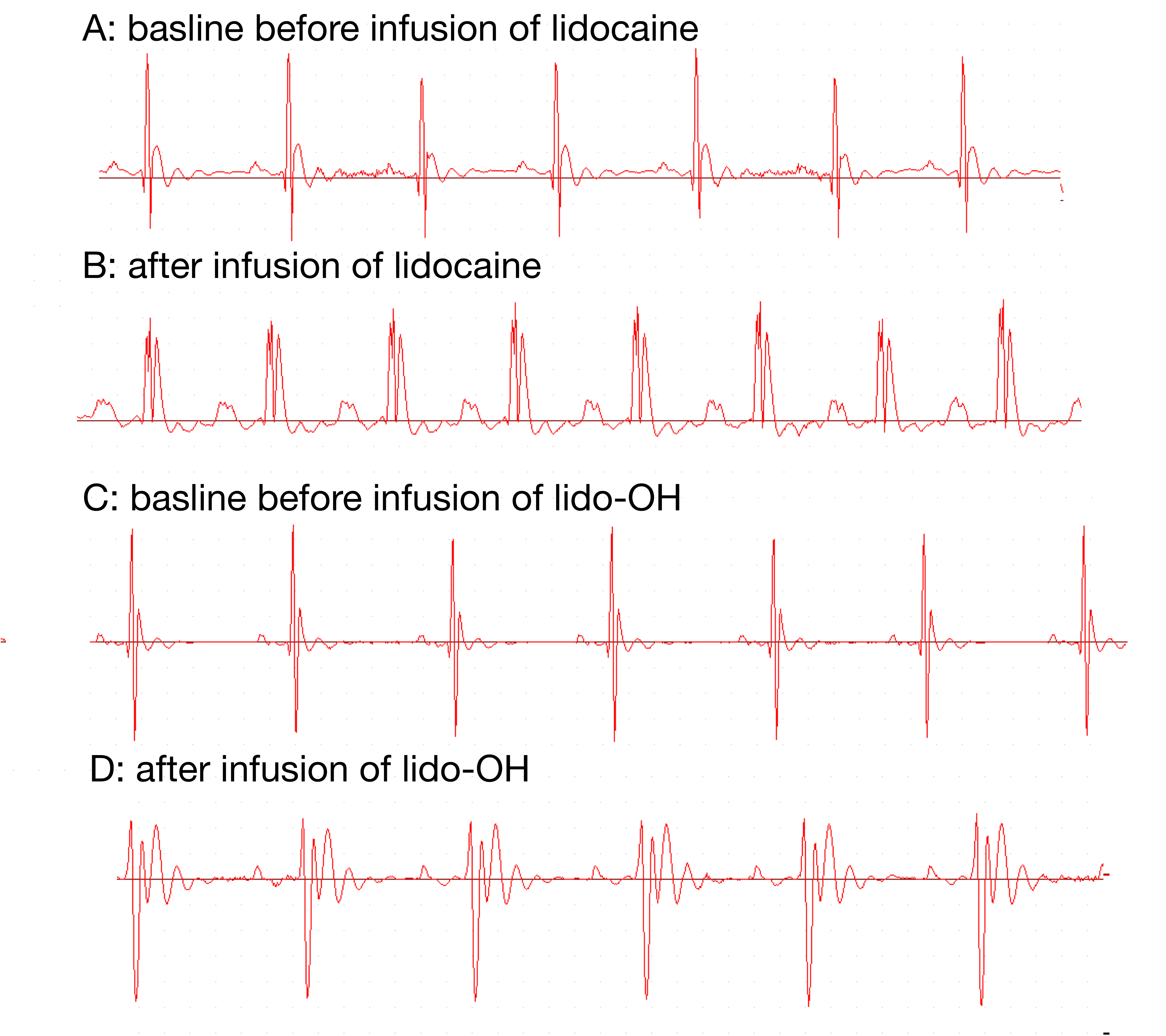


**Fig S2**. Featured electrocardiogram (ECG) of rats before and after (self-controlled) receiving intravenous infusion of high dose of lidocaine (3.5 mg/kg) or lido-OH (25.0 mg/kg) at the same infusion speed under isoflurane inhaled anesthesia. Note that drug-infusion resulted in extended P-R interval, prolonged-QRS complex, distortion of QRS wave form, and decrease in heart rate. The ED_50_ for lidocaine and lido-OH in producing positive ECG change (either HR, P-R interval, or QRS-wave length change ≥ 30% of baseline) were 3.0 mg/kg and 16.0 mg/kg, respectively.


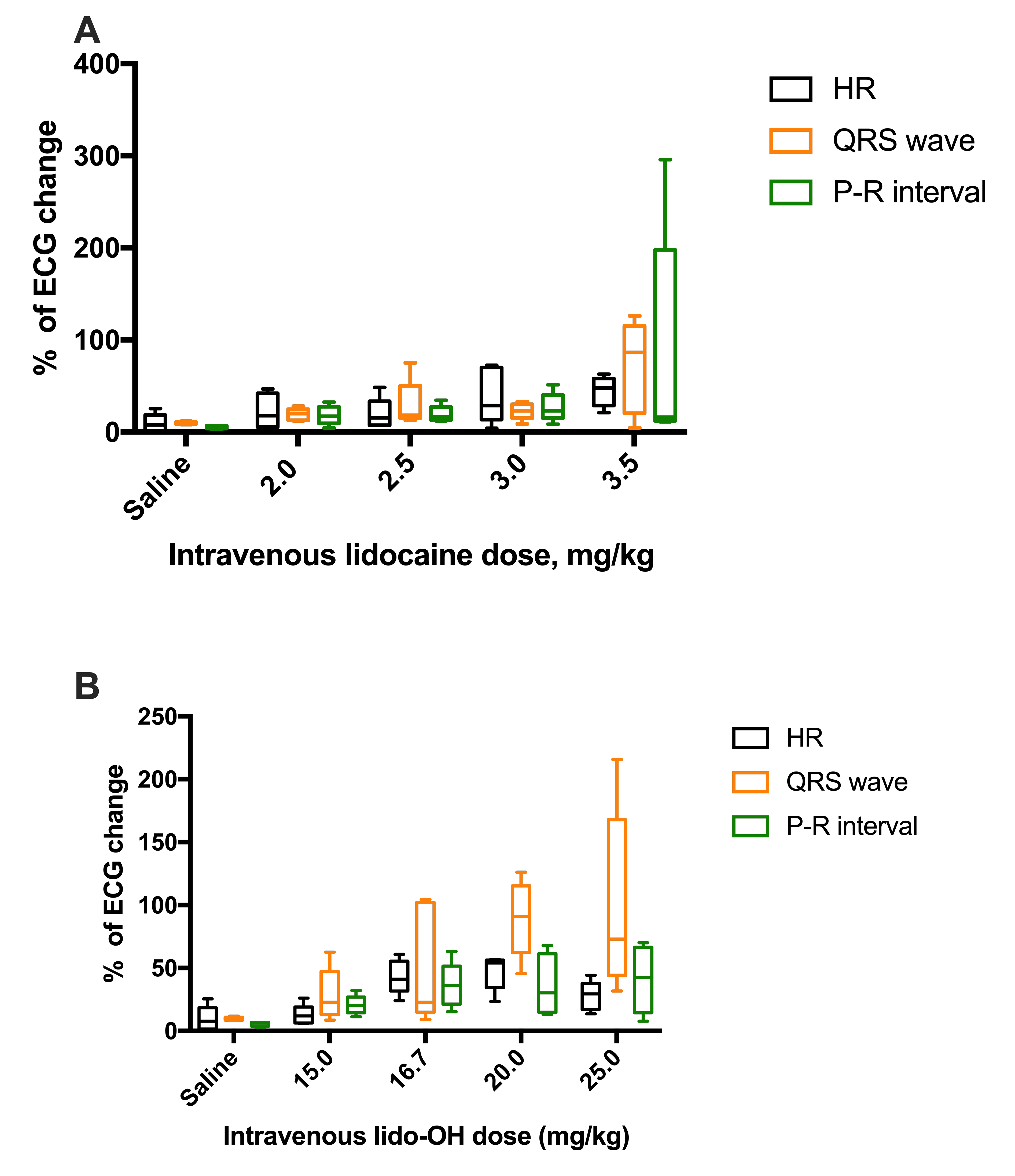


**Fig S3**. Dose-dependent electrocardiogram (ECG) change for intravenous lidocaine (A) or lido-OH (B) in isoflurane-sedated mice (n=10).
